# Level and Correlates of Unmet Need of Contraception among Women in Extended Postpartum in Northern Ethiopia

**DOI:** 10.1155/2019/6351478

**Published:** 2019-05-02

**Authors:** Gurja Embafrash, Wubegzier Mekonnen

**Affiliations:** ^1^Addis Ababa Regional Health Bureau, Ethiopia; ^2^School of Public Health, College of Health Sciences, Addis Ababa University, Ethiopia

## Abstract

**Background:**

About 222 million women in developing countries had unmet need for contraception. Women in their first year after childbirth had the largest proportion of unmet need for contraception. This first year after delivery is described as an extended postpartum period.

**Objective:**

To determine the level and correlates of unmet need for family planning among women who are in an extended postpartum period in the Tahtay Koraro District, Northern Ethiopia.

**Material and Method:**

A cross-sectional facility-based study complemented by in-depth interview of key informant was implemented. A total of 409 women in the 1st year after delivery were recruited. The study period was from 1st February to March 30, 2014. For quantitative data Epi-Info version 3.5.4 software was used for data entry, and then data were exported to SPSS Version 21 software for further analysis. Logistic regression model was used to identify factors associated with the outcome variable. The transcribed and translated qualitative text data were imported into an Open Code program and coded. Then codes were categorized and thematically described.

**Results:**

The overall unmet need for family planning was 150 (36.7%), with 121 (29.6%) for spacing and 29 (7.1%) for limiting. One hundred twenty (29.3%) women were using family planning and 94 (78.3%) of them were using injectable. The commonest reasons for nonuse of FP were nonmenstruating since last birth 201 (69.6%), side effects 39 (13.5%), and not having sex 25 (8.7%). Rural residence (AOR=7.16, 95% CI 2.57-19.95), postpartum week (26-38 weeks; AOR=8.16, 95% CI 4.24-15.71), and low perceived risk of pregnancy (AOR=1.79, 95% CI 1.04-3.09) were significantly associated with high unmet need. Opposition from different groups of the community, low perceived risk of pregnancy, provider refusal of removal of implants, and misunderstanding of FP use and side effects were additional triggering factors for unmet need.

**Conclusion and Recommendation:**

The unmet need for family planning was high. Rural residence, increased maternal postpartum week, and low perceived risk of pregnancy were associated with high unmet need. Opposition from different sects of the community and provider refusal of implant removal were also other factors triggering unmet need. Empowering women with knowledge of the risk of pregnancy and FP use during an extended postpartum period should be enhanced. Further awareness creation should be extended to periphery at different levels of the community.

## 1. Background

In 2013 the world population reached 7.2 billion, with 5.9 billion (82.5%) living in the less developed regions [1]. This hinders the reduction of poverty and the achievement of internationally agreed goals like universal access to reproductive health. And an unmet need for family planning in the least developed countries remains high [2].

Extended postpartum unmet need for family planning (FP) is the percentage of women (ages 15 to 49) within the first year following the birth of their most recent child who desire to either stop or postpone childbearing who are at risk or have returned to fertility but are not currently using a contraceptive method [3].

In 2012, 222 million women in developing countries had unmet need for modern family planning methods [4]. In sub-Saharan Africa, 24% of married women had an unmet need for contraceptive, being the lowest in Zimbabwe (13%) and the highest in Rwanda (38%) [5]. One of the main factors that account for future population growth is the increased number of unwanted births as a result of unmet need for contraception [6], which account for 79% of all unintended pregnancies in developing countries [7].

A study has shown that of all unmet needs, women in the extended postpartum period account large proportion [8]. Globally, nearly 65% of women in their first postpartum year had an unmet need for family planning services [9]. Study in Ethiopia has indicated that 86% of women in the first year postpartum have unmet need [10]. This critical period of postpartum is important to the health of both the mother and the child, and to use FP to prevent unintended pregnancy [11].

According to the Ethiopia Demographic and Health Survey (EDHS), 25% of currently married women have an unmet need for family planning—16% for spacing and 9% for limiting [12]. Community-based survey in Bahir Dar, Ethiopia, showed 21.2% women in the extended postpartum period (EPPP) had unmet need for FP [13]. However, there is a limitation of further detailed studies concerning the level and correlates of unmet need for family planning concerning women who are in this critical postpartum period in Ethiopia.

## 2. Material and Method

An institution based cross-sectional survey complemented by a qualitative study was conducted from February 1, 2014, to March 30, 2014, in the Tahtay Koraro District, Tigray regional state, Northern Ethiopia. The total population of the District was 116273, of which the female population accounts 59929 (51.5%) [14]. There were 6 health centers and 1 zonal hospital within the District.

A sample of 422 postpartum married women of reproductive age group, who were in their 1st year after delivery were included in the quantitative study. The sample size was determined using single population proportion formula, using the prevalence (51.8%) of unmet need for family planning in women of Bahir Dar, Ethiopia, who were in extended postpartum period [13]. The degree of precision was taken to be 5%, 95% confidence level, and adding 10% nonresponse rate.

The public health facilities with immunization and child health services of the study area, Tahtay Koraro District, and client flow to the respective services were identified. Then, all of the rural health centers (which were 4 in number) and the urban health centers (which were 2 in number) were selected. The sample size was assigned to each selected health facility proportional to the number of infants served to immunization and child health care services in the respective health facility. The mothers of those infants were consecutively recruited during the mornings of data collection time according to their order of arrival during their visit. Recruitment was continued until the required sample size was obtained from each facility.

The inclusion criteria were postpartum married women who were within the first year after delivery, who came for immunization and child health services with their infants (less than one-year baby) to a health center. Women with seriously ill infants and having communication problems were excluded from the study.

On the other hand, for the qualitative part of the study, an in-depth interview was conducted among those experienced and volunteer key informants residing in the selected health facilities. They were selected based on purposive sampling and an in-depth interview was continued with each informant until a point of saturation of information was reached. Consequently 6 participants who were picked from family planning programmers, health providers, and women affairs office were interviewed. The interviewees were as follows: one 60-year-old religious leader, two family health programmers (one from urban and one from rural), one health extension worker, and two long serving health providers.

Data were collected using a structured questionnaire adopted and modified from Demographic and Health Survey (DHS) which included demographic information, full details of postpartum maternal unmet need, and other factors that deter use of family planning among mothers. The questionnaire was initially prepared in English after an extensive search and review of relevant studies done on the issue under caption and then it was translated into Tigrigna, the local language and mother tongue of the study women. The Tigrigna version was again translated back to English to check for consistency of meaning of questions included in the data collection. The translated Tigrigna version questionnaire was pretested in similar areas outside of the study site prior to the actual data collection

Six data collectors and one supervisor who had a diploma in health-related fields were employed. They were trained for one day on the study instruments and data collection procedures. Data collection was carried out using a face-to-face interview with the study participants.

The data collection instruments were coded and data were checked and entered using Epi-Info version 3.5.4. It was cleaned and edited by running frequencies and cross tabulation. For analysis, the data were exported from Epi-Info to SPSS Version 21. Descriptive statistics and numerical summary measures are presented using frequency distribution tables to describe the study population in relation to relevant variables. The outcome variable, i.e., postpartum unmet need, was calculated prospectively based on future fertility preferences (looking forward) and FP use of women who were in the extended postpartum period and those who were on Lactational Amenorrhea (LAM) have been included as contraceptive users in the calculation, despite they were practicing unknowingly. This is because it is most likely to associate with the need for FP in the extended postpartum period. Since the women's return to fertility varies and it is difficult to predict its return, all postpartum women who are not using a method could be considered to have unmet need for FP [15].

Bivariate logistic regression analysis with using odds ratio along with their 95% confidence interval was used to assess the degree of association between dependent and independent variables and to test the significance of the association, respectively. Independent variables which had significant association with the outcome variable at the bivariate level were entered into multivariate analysis. Multivariate logistic regression model using adjusted odds ratio (AOR) was applied to identify the important determinants for unmet need for family planning of women who were in extended postpartum period and used to control for possible confounding effects.

For the qualitative part of the study, the tape-recorded audio data were transcribed to Tigrigna and translated to English. Open code software was used to code and categorize qualitative data, and then content analysis was employed to analyze the qualitative data. The exported raw data in open code was read text by text thoroughly and codes were labelled. After that codes were categorized into different categories. Then every category had been explained to conceptualize the interpretations of the whole data using the raw data. And a theme which fits all the categories was formulated. Finally, the findings were triangulated with the quantitative result during write-up.

An ethical approval from a research ethics committee of School of Public Health in Addis Ababa University (AAU) was obtained. Following the endorsement by the research ethics committee, Tahtay Koraro District health office was informed about the study through a support letter from School of Public Health, AAU. Accordingly, written cooperation supportive letter was prepared to the respective health facilities where the study was conducted. Informed verbal consent was obtained from each selected study woman who was in extended postpartum period and key informant to confirm willingness. The privacy and confidentiality of information have been maintained. Besides, information on available family planning methods to women in postpartum was given as direct benefits to study participants.

## 3. Results

### 3.1. Sociodemographic Characteristics

 See Table 1.

### 3.2. Reproductive History and Preferences

A total of 336 (82.2%) women had less than 5 living children while 248 (60.6%) respondents reported more than 4 ideal numbers of children. Three hundred twelve (76.3) study subjects were counseled for family planning during their contact with providers since their last birth and 97 (23.7%) were denied of it during their postpartum period (Table 2).

### 3.3. Breast Feeding, Menses, and Sexual Resumption

**Figure 1:**
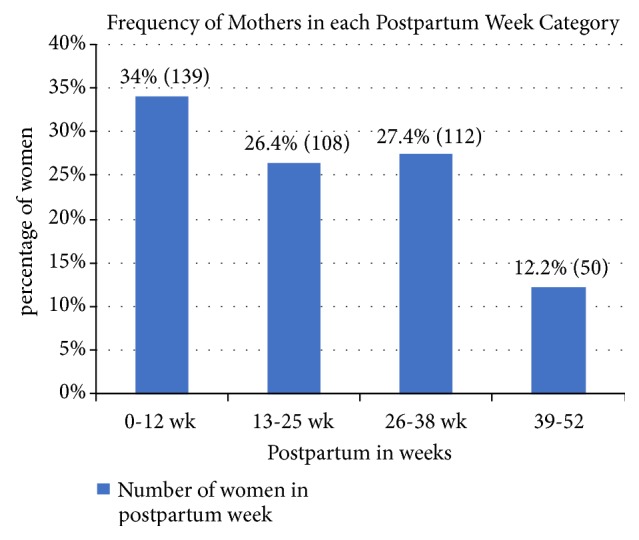
Percentage of women with respect to postpartum week in Tahtay Koraro, Ethiopia, April 2014.

All study participants were breastfeeding their children at the time of the survey. One hundred thirty-nine (34%) were in 0-12 weeks postpartum and 108 (26.4%) were in 13-25 weeks since their last birth (Figure 1).

**Figure 2:**
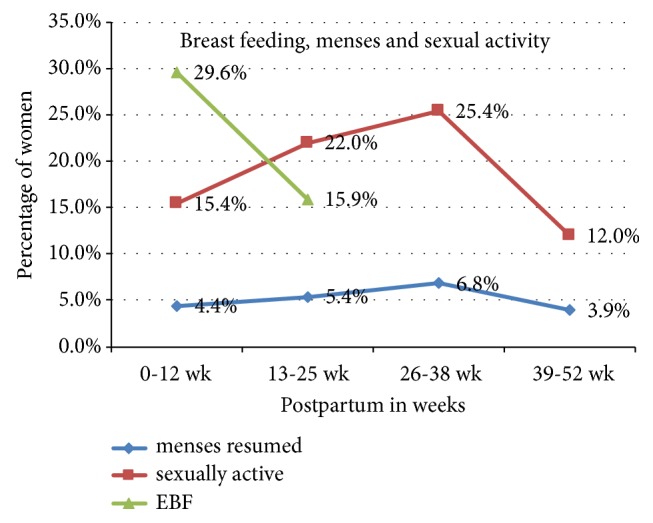
Exclusive breastfeeding, resumption of menses, and sexual activity of women in extended postpartum period in Tahtay Koraro, Ethiopia, 2014.

In most of the women in the study, 306 (74.8%) were sexually active. One hundred eighty-six (45.5%) were exclusively breastfeeding their children. Only 84 (20.5%) of all respondents reported resumption of menses. The sexual activity and the resumption of menses seem lower at 39-52 weeks postpartum, but this is due to the lower number participants in the group compared to the other postpartum week categories (Figure 2). Of all the study subjects 107 (26.2%) mothers were on Lactational Amenorrhea.

### 3.4. Knowledge, Attitude, and Approval of Family Planning Characteristics

Three hundred ninety-eight (97.3%) of the study participant knew at least one method of family planning. Two hundred seventy-nine (68.2%) women discussed about family planning with their husbands and 258 (92.5%) of them were supported to use FP by their husbands.

### 3.5. Unmet Need and Reasons for Nonuse of Family Planning Practice during the Postpartum Period

Table 3 shows the FP practice and unmet need for FP levels among the study participants. The major reason for not using family planning method was fear of side effects 30 (37%), followed by infrequent sex 14 (17.3%), and nonmenstruating since last birth 11 (13.6%). Regarding the most important part of this result which is the current status of postpartum unmet need (prospective unmet need); 257 (62.8%) of women were not using family planning despite the fact that they need to space or limit their births. However, 107 (26.2%) women were practicing Lactational Amenorrhea unknowingly. Thus 150 (36.7%) women had actual unmet need with 121 (29.6%) for spacing and 29 (7.1%) for limiting.

The dominant reason for current nonuse of family planning methods were as follows: nonmenstruating since last birth 201 (69.5%), fear of side effects/ health concerns 39 (13.5%), infrequent sex 22 (7.6%), and husband's opposition 15 (5.2%). This could be complemented by the findings of qualitative data.

A 46-year-old male long serving health provider who is a FP programmer from the district health office said:* “The most prevalent reason is that they don't know when pregnancy can happen to them. They say ‘as long as menses is not resumed and we are breastfeeding for one- or two-years pregnancy can't happen. So why do we need to suffer by taking drugs while we are breastfeeding our children and preventing pregnancy through that.'”*

Another 28-year-old health extension worker also added an important issue and she said: “Another factor is about the long acting Implanon. We are trained to insert but not to remove. When we refer mothers to a health center or hospital for possible removal after complaints of side effects or others, they refuse to remove before its time frame (required time) which is 3 years. Then they return back to our facility for complaining and shout at us why we insert it if we can't remove it. And they say ‘they can only insert but they can't remove'. This issue has created some sort of disappointment in using long acting FP in our community.”

**Figure 3:**
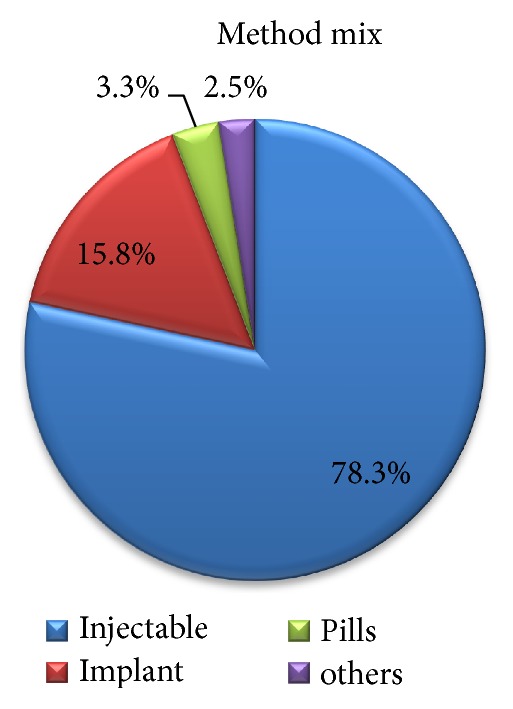
Method mix of women in extended postpartum period in Tahtay Koraro, Ethiopia, 2014.

 Of the 409 study participants, only one hundred twenty (29.3%) women were using family planning. Of which 94 (78.3%) and 19 (15.8) of them were using injectable and implant, respectively (Figure 3).

### 3.6. Factors Affecting Unmet Need in Women Who Are in Extended Postpartum Period

In the multivariate analysis, maternal residence, maternal postpartum week, and perceived risk of pregnancy were the only factors which were significantly associated with the postpartum unmet need (Table 4). Accordingly, having all variables controlling the odds of women who resided in rural area had 7 times more likely to have unmet need compared to those who were urban dwellers (*p*-value.000; AOR 7.16; 95% CI (2.57 -19.95)). Those women who were in their 39-52 postpartum weeks were 8.7 times more likely to have unmet need compared to those who were at their 0-12 weeks postpartum (P-value.000; AOR 8.71; 95% CI (3.90-19.44). And women who had low perceived risk of pregnancy were 1.8 times more likely to have unmet need in relation to those who had high perceived risk of pregnancy (p-value.037; AOR 1.79; 95% CI (1.04-3.09)).

## 4. Discussion

This study has investigated the magnitude and correlates of postpartum unmet need among married women during the extended postpartum period. Accordingly, using the prospective approach of unmet need, the study revealed that the unmet need for family planning of women who were in the 1st year after delivery was 36.7% with 29.6% for spacing and 7.1% for limiting. The rate of unmet need found in this study was lower than the study done in Ethiopia, Nigeria, and Tanzania [10, 16, 17]. The possible reasons for the variations might be due to the expanding health services coverage and increased awareness of FP and maternal health services in the study area in particular, and Ethiopia in general. The Nigerian study calculation was based on the intentions to postpone childbearing by at least 6 months from the time of the survey, while this study was designed at their wish to delay the next pregnancy for a minimum of two years. The Ethiopian study was based on EDHS analysis for which the variations might be attributed to its multicultural and religious group's inclusion, which could possibly increase the unmet need. In addition, it was higher than the study done in Bahr Dar, Ethiopia [13]. The variation was due to setting difference, as the study from Bahir Dar was done in urban settings where unmet need in urban areas of Ethiopia is lower than the rural [12, 13]. But this study has included both urban and rural populations.

Our results showed that rural residents were 7 times more likely to have unmet need compared to those urban dwellers (AOR 7.16; 95% CI (2.57-19.95)) which is consistent with the EDHS, 2011. This might be due to limited access to FP services in rural settings. Higher postpartum insusceptibility of rural women than urban women to pregnancy associated with longer duration of breastfeeding in rural women [18] could also be another factor. Because the delaying effect of breastfeeding on menses could make them feel insusceptible to pregnancy. This could further be strengthened by the finding that 95 (63.3%) of mothers who had unmet need were women from the rural areas who thought pregnancy would not happen unless menses is resumed and only 13 (8.7%) of urban women had unmet need who had a similar perception.

The postpartum unmet need was more likely to increase as the postpartum week increased. Women in their 26-38 weeks postpartum were 8.2 times more likely to have unmet need compared to those who were on their 0-12 weeks postpartum (AOR 8.16; 95% CI (4.24-15.71)). The result also showed that unmet need for FP remained high throughout the 1st year after delivery, which is consistent with the EDHS 2005 based study analysis [10]. This can be explained by the fact that the contribution of LAM to protect pregnancy is diminished as postpartum period increases. By 7-9 months after birth, most women become exposed to pregnancy. Though they do not want to become pregnant again so soon, they still do not get contraceptive protection and this leads them to have a high unmet need [8]. Moreover, prolonged breastfeeding could possibly delay menses for which many women make it as a benchmark to utilize contraceptive. And they believe that they cannot get pregnant unless their menses have returned, which is not true [19].

Women with low perceived risk of pregnancy (LPRP) (i.e., women who thought that pregnancy would not happen if menses is not resumed) were 1.8 times more likely to have unmet need compared to those who had high perceived risk of pregnancy (HPRP) (i.e., women who thought that pregnancy could happen though menses is not resumed) (AOR 1.79; with 95 CI (1.04-3.09)). This could be explained by the result that of the 289 nonusers of FP, 211 (73%) of them had the perception that pregnancy would not happen if menses is not resumed. This is consistent with Cleland et al. (2016) study that for some women, low perceived risk of pregnancy is the main reason for unmet need [6]

Although most respondents were able to know at least one method of FP, less than one-third of all participants were using FP. This was consistent with the study from Nigeria and Democratic Republic of Cong [16, 20]. This low uptake of family planning could be explained by the fact that resumption of menses was a strong factor affecting utilization of contraceptive in the postpartum period [21]

Of the 289 nonusers of FP, 201 (69.9%) mentioned nonmenstruating since their last birth as a dominant factor for their nonuse of FP. However, a woman may ovulate before the 1st menstruation following a birth and the risk of pregnancy preceding 1st menstruation increases as the postpartum duration increases [15]. A study in Egypt confirmed that out of the 4.4% pregnancy occurred in the first 6 months, 15.1% of pregnancy occurred before resumption of menses, and 28.1% occurred while they were exclusively breastfeeding [22]. Other reasons were fear of side effects 39 (13.5%), not having sex 25 (8.7%), and infrequent sex 22 (7.6%), which is consistent with the study done in Rwanda [23]

This study has tried to complement the quantitative data by a qualitative method. And it included both urban and rural study participants. So, the implementation of mixed method could be the strong side of the study. Since the study was facility-based, generalization of the findings to the overall population of the study area is difficult. Women who were on LAM were considered as contraceptive users despite the fact that they were practicing unknowingly and the proportion of exclusive breastfeeding was assessed using previous day recall. This would probably cause the proportion of exclusively breastfed infants to be overestimated, which in turn may increase LAM users. The inability of identifying the actual users of LAM was also a weakness of this study. Failure to include women of the study as key informant can also be considered as a drawback. However, to maximize the validity of the study, we have used possible maximum study participants. In addition to this, the proper data collection method and uniformly structured questionnaires were used along with the deployment of well-trained data collectors. Beside this, qualitative data were collected from key informants till the point of saturation was achieved.

## 5. Conclusion and Recommendation

This study has revealed that the prevalence of unmet need during the extended postpartum period was significantly higher. The sizeable proportion of women who wanted to space or limit their births in this critical period were not using a contraceptive method, even though some were protected by Lactational Amenorrhea unintentionally.

Rural residence, maternal postpartum week and low perceived risk of pregnancy were found to be the determinants of the postpartum unmet need during the extended postpartum period. And all were associated with increased occurrence of unmet need.

Though most women of the study had the knowledge of at least one FP method, only 29.3% of them were using FP. The dominant reason for current nonuse of family planning was being nonmenstruated since last birth, followed by fear of side effects, infrequent sex, and having no sex, respectively. At the same time qualitative findings of key informants of this study revealed that low perceived risk of pregnancy, fear of infertility, opposition from different sects of the community, provider negligence, refusing removal of implants, misunderstanding of FP use and side effects, and poor competence of health extension workers were among the dominant factors raised that hinder FP utilization of women who are in the extended postpartum period.

Rural postpartum women should be aware of the risk of pregnancy during the first year after delivery and should be encouraged to use appropriate family planning method to increase utilization. They should be given special attention in acquiring knowledge on FP use and its side effects as well. Particularly, postpartum women and health care providers ought to be alert that pregnancy could occur before the resumption of menses after delivery, and women should not wait the resumption of menses to use FP.

The capability of health extension workers in the provision of insertion and removal of long acting FP should be strengthened. Health care providers should work hard to alleviate the negative influence of religious leaders, mother in-laws, and husbands' oppositions towards the use of family planning methods. While health care providers counsel postpartum women on FP use and risk of pregnancy, they should also give emphasis on the appropriate use of the LAM method up to six months of postpartum. Health extension workers also need to be informed about LAM too that Lactational Amenorrhea is a reasonably good contraceptive method only up to six months of postpartum and that every woman should be using another method since then on. Mothers should particularly be counseled about those things during their contacts in antenatal care, postnatal care, immunization, and child health services.

Health Extension workers should get proper training on removal as well if they get training on insertion. And they should not be allowed to do insertion without the capacity to remove the implants. At the same time, they should be trained in implant's counseling with great emphasis in explaining the bleeding disturbance that implant's users will have for six months or longer after insertion. This is to make sure that no woman is receiving an implant without being fully aware of the major side effects.

A nationally representative study involving diversified communities in the country is recommended. Especially community-based comparative study complemented with qualitative data would be helpful.

## Figures and Tables

**Table 1:** Sociodemographic characteristics of women in extended postpartum period, in Tahtay Koraro Woreda, Ethiopia, 2014*. (N=409)*.

Variables		Frequency	Percent (%)
Maternal age	15-19	49	12
	20-24	115	28.1
	25-29	106	25.9
	30-34	71	17.4
	35 and above	68	16.6

Maternal residence	Urban	135	33
	Rural	274	67

Maternal religion	Orthodox	377	92.2
	Muslim	32	7.8

Maternal education	No education	176	43
	Primary	143	35
	Secondary and above	90	22

Maternal occupation	Housewife	112	27.4
	Farmer	241	58.9
	Merchant	36	8.8
	Others*∗*	20	4.9

Husband's education	No education	105	25.7
	Informal education	28	6.8
	Primary	156	38.1
	Secondary and above	112	27.4
	Others (Unknown)	8	2.0

Husband's occupation	Farmer	245	59.9
	Government employee	41	10
	Merchant	53	13
	Daily laborer	70	17.1

Household monthly income	Low income (663 and below EBR)	207	50.6
	High income (>663 EBR)	202	49.4

(*∗* = students, government employee and daily laborer).

**Table 2:** Reproductive health characteristics of postpartum women in Tahtay Koraro Woreda, Tigray, Ethiopia, 2014. *(N=409)*.

Variable		Frequency	Percentage (%)
No. of living children	<5	336	82.2
	5 and above	73	17.8

Parity	1	121	29.6
	2-3	145	35.5
	4 and above	143	35

Ideal N**o**. of children	<5	161	39.4
	5 and above	248	60.6

ANC visits	Yes	390	95.4
	No	19	4.6

Ever use of FP method	Yes	239	58.4
	No	170	41.6

FP counselled	Yes	312	76.3
	No	97	23.7

**Table 3:** Unmet need for FP, FP use, and reasons for nonuse of FP in EPPP, in Tahtay Koraro, Tigray, Ethiopia, 2014.

Variables	Frequency	Percentage (%)
Reasons for non-use of FP (retrospective) (n=81)		
Fear of side effects/ health concerns	30	37
Infrequent sex	14	17.3
Non-menstruating since last birth	11	13.6
Up to God	11	13.6
Husband opposition	6	7.4
Others	11	13.6

Current FP use		
Yes	120	29.3
No	289	70.7
Total	409	100%

Unmet need with LAM exclusion (prospective)		
Spacing	207	50.6
Limiting	50	12.2
Total	257	62.8%

Unmet need (prospective, outcome variable)		
Spacing	*121*	*29.6*
Limiting	*29*	*7.1*
Total	*150*	*36.7*%

Reasons for current non-use of FP (n=289)		
No-menstruating since last birth	201	69.6
Side effects/health concerns	39	13.5
Not having sex	25	8.7
Infrequent sex	22	7.6
Husband opposition	15	5.2
Breast feeding	13	4.5
Want another child	8	2.8
Up to God	7	2.4
Others	11	3.8

(NB: for the reason of non-FP use, n=81 and n=289, the total sum of the reasons is higher because some women gave more than one reason as multiple response was allowed).

**Table 4:** The Association between unmet need for FP and different characteristics of women who are in EPPP in Tahtay Koraro Woreda, Tigray, Ethiopia, 2014.

Variables	Total unmet need		Crude odd ratio	Adjusted odds ratio
	Yes	No	95% CI	95% CI
Maternal age				
15-19	14	35	1	1.00
20-24	41	74	1.39 (.67-2.87)	1.77 (.75-4.20)
25-29	38	68	1.40 (.67-2.92)	2.28 (.94-5.56)
30-34	24	47	1.28 (.58-2.82)	1.54 (.59-4.02)
35-49	33	35	2.36 (1.08-5.15) *∗*	1.82 (.68-4.88)
Maternal residence				
Urban	24	111	1.00	1.00
Rural	126	148	3.94 (2.39-6.50) *∗∗*	7.16 (2.57-19.95) *∗∗*
Maternal education				
No education	78	99	1.00	1.00
Primary	52	90	.73 (.47-1.15)	1.15 (.62-2.13)
Secondary and above	20	70	.36 (.20-.65) *∗*	.79 (.33-1.88)
Maternal occupation				
Housewife	32	80	1.00	1.00
Farmer	108	133	2.03 (1.25-3.29) *∗*	.47 (.80-2.80)
Merchant	8	28	.71 (.294-1.73)	.78 (.25-2.48)
Others^**#**^	2	18	.28 (.06-1.27)	.27 (.05-1.61)
Husband's education				
No education	42	63	1.00	1.00
Informal education	11	17	.97 (.41-2.28)	.68 (.26-1.80)
Primary	69	87	1.19 (.72-1.97)	1.41 (.78-2,57)
Secondary and above	27	85	.46 (.27-.85) *∗*	1.44 (.63-3.30)
Husband's occupation				
Farmer	110	135	1.00	1.00
Government employee	9	32	.35 (.16-.75) *∗*	1.10 (.15-7.97)
Merchant	12	41	.36 (.18-.72) *∗*	.89 (.14-5.83)
Daily laborer	19	51	.46 (.26-.82) *∗*	.67 (.10-4.31)
Household income				
Low income	24	111	1.00	1.00
High income	126	148	.65 (.44-.98) *∗*	1.16 (.67-2.02)
Maternal PP week				
0-12	23	116	1.00	1.00
13-25	35	73	2.42 (1.32-4.42) *∗*	2.78 (1.43-5.40) *∗*
26-38	63	49	6.49(3.6211.61) *∗∗*	8.16 (4.24-15.71) *∗∗*
39-52	29	21	6.97(3.40 14.28) *∗∗*	8.71 (3.90-19.44) *∗∗*
Ideal No. of children				
Less than 5	42	119	1.00	1.00
5 and above	108	140	2.19 (1.42-3.37) *∗∗*	1.18 (.66-2.13)
Perceived risk of pregnancy while menses absent				
Yes (high perceived risk)	42	125	1.00	1.00
No (low perceived risk)	108	134	2.40 (1.56-3.69) *∗∗*	1.79 (1.04-3.09) *∗*

*∗* stands for *p*-value < .05 and *∗∗* for p-value < .001. ^**#**^Students, government employee, and daily laborer.

## Data Availability

The data used to support the findings of this study are included within the article.
